# Supplementary material for: Detection of Immune Escape and Basal Core Promoter/Precore Gene Mutations in Hepatitis B Virus Isolated from Asymptomatic Hospital Attendees in Two Southwestern States in Nigeria
Source: Viruses. 2023 Oct 31;15(11):2188. doi: 10.3390/v15112188 (PMC10674980; doi:10.3390/v15112188)
Supplement: Supplementary file 1 [file viruses-15-02188-s001.zip › viruses-2657700-supplementary.pdf]

Supplementary Table S1. Prevalence of HBV biomarkers by socio-demographic characteristics of participants

Variables	HBsAg Positive (%)	HBsAb Positive (%)	Anti-HBV IgM Positive (%)	Anti-HBc Positive (%)	HBeAg Positive (%)	HBeAb Positive (%)
State						
Ekiti	20(13.3%)	70(46.6%)	6(4%)	71(47.3%)	7(4.7%)	25(16.7%)
Osun	31(11.9%)	93(35.7%)	2(0.7%)	49(18.8%)	5(1.9%)	28(10.7%)
**Age (Years)**						
≤24	3(7.3%)	17(41.5%)	0(0%)	17(41.5%)	0(0)	5(12.2%)
25-34	18(11.9%)	56(37.1%)	3(2%)	47(31.1%)	5(3.3%)	14(9.3%)
35-44	18(15.3%)	47(39.8%)	4(3.4%)	30(25.4%)	5(4.2%)	21(17.8%)
≥45	12(12%)	43(43.0%)	1(1%)	26(26%)	2(2%)	13(13%)
**Sex**						
Male	19(15%)	57(44.9%)	1(0.8%)	35(27.6%)	5(3.9%)	15(11.8%)
Female	32(11.3%)	106(37.5%)	7(2.5%)	85(30%)	7(2.5%)	38(13.4%)
**Marital status**						
Single	17(17%)	38(38%)	1(1%)	28(28%)	1(1.1%)	13(13%)
Married	34(11%)	124(40.3%)	7(2.3)	91(29.5%)	11(3.6%)	40(13%)
Divorced	0(0%)	1(50%)	0(0)	1(50%)	0(0)	0(0)
**Educational status**						
No formal	6(10.5%)	38(38%)	2(3.5%)	18(31.6%)	1(1.8%)	7(12.3%)
Secondary	12(9.5%)	124(40.3%)	2(1.6%)	40(31.7%)	3(2.4%)	12(9.5%)

Undergraduate	5(11.6%)	1(50%)	0(0)	14(32.6)	0(0)	4(9.3%)
Graduate	28(15.2%)	38(38%)	4(2.2%)	48(26.2%)	8(4.3%)	3(16.3%)
**Occupation**						
Unemployed	4(7.1%)	24(42.9%)	17(30.4%)	17(30.4%)	1(1.8%)	6(10.7%)
Self-employed	26(11.8%)	88(39.8%)	4(1.8%)	70(31.7%)	5(2.3%)	25(11.3%)
Civil-servant	21(15.8%)	51(38.3%)	4(1.3%)	33(24%)	6(4.5%)	22(16.5%)

Supplementary Table S2: Demographic data and molecular results of HBsAg-positive cohorts analyzed in this study

S/N	Sample ID	Study Population	Age	Gender	Location	HBeAg Status	PCR Outcome<br>S gene	 BCP/PC region
1	EK37	Blood Donor	47	M	Ekiti	Neg	Pos	Pos
2	OS155	Out-patients	35	M	Osun	Neg	Pos	Pos
3	OS182	Perinatal	49	F	Osun	Neg	Pos	Pos
4	OS239	Perinatal	27	F	Osun	Neg	Pos	Pos

5	EK68	Blood Donor	47	M	Ekiti	Neg	Pos	Pos
6	OS242	Out-patients	39	F	Osun	Pos	Pos	Pos
7	EK101	Blood Donor	32	M	Ekiti	Neg	Pos	Pos
8	OS216	Out-patients	35	M	Osun	Neg	Pos	Pos
9	OS245	Blood Donor	46	M	Osun	Neg	Pos	Pos
10	OS249	Blood Donor	43	M	Osun	Neg	Pos	Pos
11	EK19	Blood Donor	33	F	Ekiti	Pos	Pos	Pos
12	EK97	Blood Donor	32	M	Ekiti	Neg	Pos	Pos
13	EK1	Perinatal	40	F	Ekiti	Neg	Pos	Pos
14	OS142	Out-patients	29	F	Osun	Neg	Pos	Pos
15	OS132	Out-patients	22	M	Osun	Neg	Pos	Pos
16	0S175	Blood Donor	49	M	Osun	Neg	Pos	Pos
17	OS147	Perinatal	22	F	Osun	Neg	Pos	Pos
18	EK124	Blood Donor	40	M	Ekiti	Neg	Pos	Pos
19	OS68	Blood Donor	46	M	Osun	Neg	Pos	Pos
20	OS2	Out-patients	35	F	Osun	Neg	Pos	Pos
21	OS3	Blood Donor	33	M	Osun	Neg	Pos	Pos
22	OS49	Out-patients	36	M	Osun	Neg	Pos	Pos
23	OS86	Perinatal	36	F	Osun	Neg	Pos	Pos
24	OS190	Out-patients	51	M	Osun	Neg	Pos	Pos
25	OS239	Out-patients	49	M	Osun	Neg	Pos	Pos
26	EK43	Blood Donor	27	M	Ekiti	Neg	Pos	Pos
27	EK4	Blood Donor	49	F	Ekiti	Pos	Pos	Pos
28	OS283	Out-patients	35	M	Osun	Neg	Pos	Pos
29	EK100	Blood Donor	22	M	Ekiti	Neg	Pos	Pos
30	OS233	Blood Donor	45	F	Osun	Neg	Pos	Pos
31	EK73	Out-patients	47	M	Ekiti	Neg	Pos	Pos
32	EK105	Out-patients	35	M	Ekiti	Neg	Pos	Pos
33	OS20	Out-patients	39	M	Osun	Pos	Pos	Pos
34	OS1	Perinatal	27	F	Osun	Pos	Pos	Pos
35	OS215	Out-patients	32	M	Osun	Pos	Pos	Pos
36	OS80	Out-patients	35	M	Osun	Neg	Pos	Pos
37	EK175	Out-patients	30	M	Ekiti	Neg	Pos	Pos

38	EK98	Perinatal	40	F	Ekiti	Neg	Pos	Pos
39	EK67	Blood Donor	25	M	Ekiti	Neg	Pos	Pos
40	EK92	Blood Donor	27	M	Ekiti	Neg	Pos	Pos
41	EK43	Blood Donor	37	F	Ekiti	Pos	Pos	Pos
42	EK180	Blood Donor	32	F	Ekiti	Pos	Pos	Pos
43	EK54	Out-patients	38	F	Ekiti	Neg	Pos	Pos
44	EK110	Blood Donor	38	F	Ekiti	Neg	Pos	Pos
45	OS243	Out-patients	31	M	Osun	Neg	Pos	Pos
46	OS41	Out-patients	25	M	Osun	Neg	Pos	Pos
47	OS31	Perinatal	30	F	Osun	Neg	Pos	Pos
48	OS36	Blood Donor	27	M	Osun	Neg	Pos	Pos
49	OS235	Blood Donor	34	F	Osun	Neg	Pos	Pos
50	OS254	Out-patients	45	M	Osun	Neg	Pos	Pos
51	OS175	Out-patients	35	M	Osun	Neg	Pos	Pos

N/B: F = Female, M = Male, Neg = Negative, Pos = Positive
